# Impaired neutrophil extracellular trap formation: a novel defect in the innate immune system of aged individuals

**DOI:** 10.1111/acel.12222

**Published:** 2014-04-30

**Authors:** Jon Hazeldine, Phillipa Harris, Iain L Chapple, Melissa Grant, Hannah Greenwood, Amy Livesey, Elizabeth Sapey, Janet M Lord

**Affiliations:** 1 MRC-ARUK Centre for Musculoskeletal Ageing Research, School of Immunity and Infection, Birmingham University Medical SchoolBirmingham, B15 2TT, UK; 2 Department of Periodontology, School of Dentistry, University of BirminghamBirmingham, B15 2TT, UK; 3 School of Clinical and Experimental Medicine, The Medical School, University of BirminghamBirmingham, B15 2TT, UK

**Keywords:** aging, neutrophil, neutrophil extracellular traps, periodontitis

## Abstract

Neutrophil extracellular traps (NETs) are a recently discovered addition to the defensive armamentarium of neutrophils, assisting in the immune response against rapidly dividing bacteria. Although older adults are more susceptible to such infections, no study has examined whether aging in humans influences NET formation. We report that TNF-α-primed neutrophils generate significantly more NETs than unprimed neutrophils and that lipopolysaccharide (LPS)- and interleukin-8 (IL-8)-induced NET formation exhibits a significant age-related decline. NET formation requires generation of reactive oxygen species (ROS), and this was also reduced in neutrophils from older donors identifying a mechanism for reduced NET formation. Expression of IL-8 receptors (CXCR1 and CXCR2) and the LPS receptor TLR4 was similar on neutrophils from young and old subjects, and neutrophils challenged with phorbol-12-myristate-13-acetate (PMA) showed no age-associated differences in ROS or NET production. Taken together, these data suggest a defect in proximal signalling underlies the age-related decline in NET and ROS generation. TNF-α priming involves signalling through p38 MAP kinase, but activation kinetics were comparable in neutrophils from young and old donors. In a clinical setting, we assessed the capacity of neutrophils from young and older patients with chronic periodontitis to generate NETs in response to PMA and hypochlorous acid (HOCL). Neutrophil extracellular trap generation to HOCL, but not PMA, was lower in older periodontitis patients but not in comparison with age-matched controls. Impaired NET formation is thus a novel defect of innate immunity in older adults but does not appear to contribute to the increased incidence of periodontitis in older adults.

## Introduction

Comprised of a DNA backbone decorated with histones and such granule-derived peptides and enzymes as myeloperoxidase (MPO), neutrophil elastase and lactoferrin (Brinkmann *et al*., 2004), neutrophil extracellular traps (NETs) are a novel feature of the defensive armamentarium of neutrophils. Generated in response to a wide range of stimuli, which include pro-inflammatory agonists [e.g. interferon-α, interleukin-8 (IL-8), lipopolysaccharide (LPS)], the pharmacological agent phorbol 12-myristate 13-acetate (PMA), as well as microbes (e.g. bacteria, fungi and protozoa) and their products (Cooper *et al*., 2013), NETs, as their name suggests, are highly efficient at pathogen entrapment. Indeed, *in vitro*, NETs have been shown to trap bacteria, parasites and fungi (Brinkmann *et al*., 2004; Urban *et al*., 2006; Guimaraes-Costa *et al*., 2009), whilst *in vivo*, increased bacterial dissemination has been observed in pathogen-infected mice administered exogenous DNases (Meng *et al*., 2012; Yipp *et al*., 2012). Interestingly, despite the fact that the granule-derived proteins resident on NETs retain their activity when bound to DNA (Parker *et al*., 2012), NETs are themselves not directly microbicidal (Menegazzi *et al*., 2012; Parker *et al*., 2012). Instead, these structures are thought to confer protection by neutralizing the virulence factors of invading microbes (Brinkmann *et al*., 2004) and via their immunomodulatory effects, which include activating the human complement system (Oehmcke *et al*., 2009) and enhancing the activity of bystander T cells (Tillack *et al*., 2012).

Three key molecular processes underlie the formation of NETs, namely reactive oxygen species (ROS) generation, autophagy and the hypercitrullination of histones H3 and H4, a process catalysed by the calcium-dependent nuclear enzyme peptidylarginine deiminase 4 (PAD4) (Remijsen *et al*., 2011). To date, attention has focused predominantly on the generation of ROS, which play an essential role in NET formation. Pretreating neutrophils from healthy subjects with inhibitors of the ROS-generating enzyme nicotinamide adenine dinucleotide phosphate-oxidase (NADPH oxidase) or the antioxidant protein taurine prevents NET formation to a wide array of stimuli (Fuchs *et al*., 2007). Similarly, when challenged with pathogens or PMA, neutrophils isolated from patients with chronic granulomatous disease (CGD), an inherited disorder in which subunits of NADPH oxidase are either absent or dysfunctional (Seger, 2008), fail to generate NETs (Fuchs *et al*., 2007; Bianchi *et al*., 2009; Nishinaka *et al*., 2011). However, NETs are produced when these cells are challenged with exogenous hydrogen peroxide (H_2_O_2_), further highlighting the importance of ROS in NET generation. H_2_O_2_, however, does not appear to be the active oxygen species that triggers NET generation, with this function attributed instead to its downstream products hypochlorous acid (HOCL) and singlet oxygen (Nishinaka *et al*., 2011; Palmer *et al*., 2012).

Compared with younger subjects, older adults report an increased incidence of bacterial and fungal infection (Kauffman, 2001; Laupland *et al*., 2003). As neutrophils represent the frontline defence against these pathogens, this increased susceptibility to infection implies that the protective nature of neutrophils wanes with age. Indeed, as a result of impairments in several defensive strategies such as phagocytosis (Wenisch *et al*., 2000; Butcher *et al*., 2001), degranulation (McLaughlin *et al*., 1986) and ROS production (Fulop *et al*., 2004), the microbicidal activity of neutrophils from older adults is markedly reduced (Wenisch *et al*., 2000; Simell *et al*., 2011). Recently, Tseng and colleagues (Tseng *et al*., 2012) made the novel observation that NET formation is also impaired with age, with the group demonstrating neutrophils from aged mice generated significantly fewer NETs when challenged with *Staphylococcus aureus* (Tseng *et al*., 2012). *In vivo*, this defect in NET generation was associated with marked bacteraemia, leading the group to hypothesize that aberrant NET formation may explain why older adults are more susceptible to invasive bacterial disease following skin and soft tissue infection (Tseng *et al*., 2012). However, the study did not investigate the impact of physiological aging on NET generation by human neutrophils or the mechanisms involved.

To address this important issue, we isolated neutrophils from healthy young and old donors and compared both NET production and ROS generation by these cells in response to IL-8, LPS or PMA stimulation. In addition, in order to provide clinical relevance to this study, we compared NET generation by neutrophils isolated from young and older patients with chronic periodontitis, a chronic infectious bacterial disease of humans that increases in prevalence with aging (White *et al*., 2012). Periodontitis is initiated by the accumulation of a pathogenic plaque biofilm at and below the gingival margin, but the resulting connective tissue damage is largely mediated by an abnormal host response, dominated by neutrophilic inflammation. Thus, neutrophils are able to migrate to the site of infection, but the inflammatory immune response fails to eliminate the pathogenic bacteria (Dias *et al*., 2013), leading to a chronic nonresolving inflammation (Van Dyke, 2008).

## Results

### Neutrophil priming significantly enhances NET production and ROS generation

In the majority of NET studies published to date, resting neutrophils have been used in NET generation assays. Although capable of generating NETs, the use of resting neutrophils does not reflect the *in vivo* state of neutrophils at times of infection when exposure to pro-inflammatory cytokines and bacterial products leads to ‘priming’, which heightens neutrophil responses and microbicidal activity. Thus, to mimic more closely the conditions under which neutrophils *in vivo* would generate NETs, we exposed neutrophils to tumour necrosis factor-alpha (TNF-α), a pro-inflammatory cytokine whose levels are increased during infection and in inflammatory states, prior to stimulation with IL-8 or LPS.

**Figure 1:**
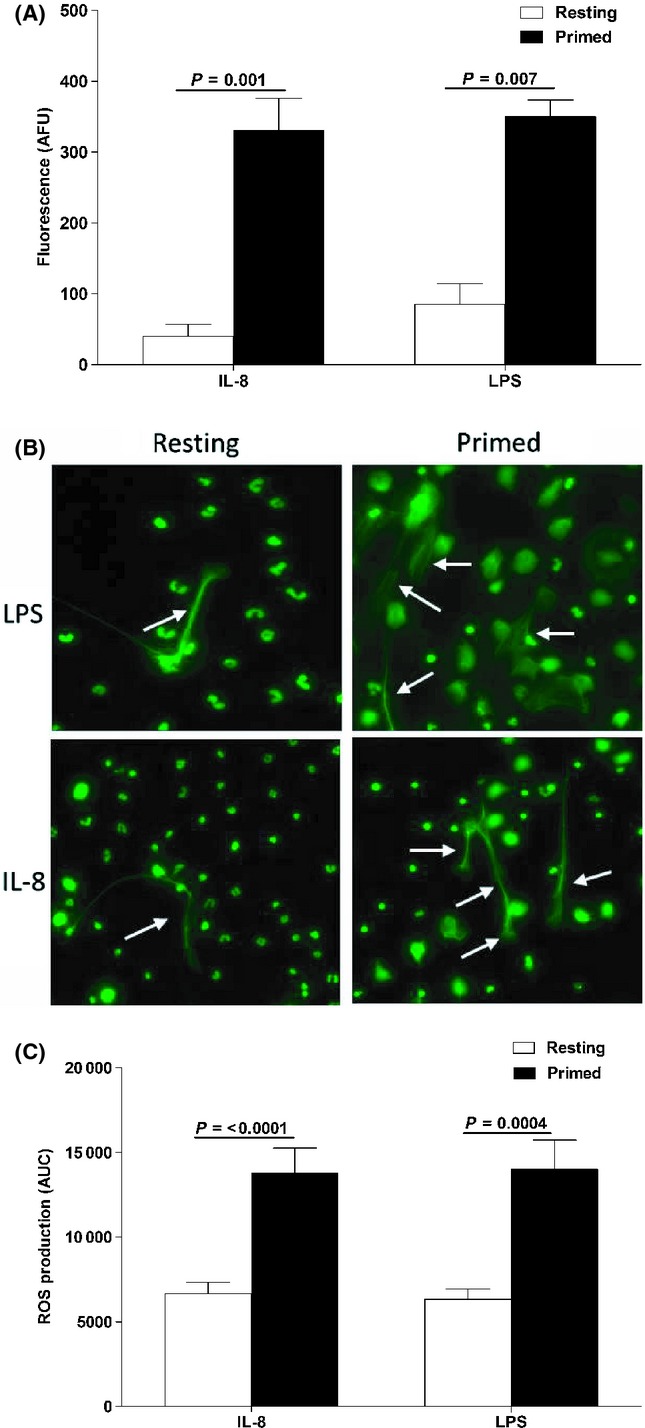
Neutrophil priming significantly increases the NET production and ROS generation. (A) Neutrophils isolated from young adults (*n* = 5) were cultured for 15 min in the presence (black bars) or absence (white bars) of 10 ng mL^−1^ TNF-α followed by a 3-h stimulation with 10 ng mL^−1^ IL-8 or 100 ng mL^−1^ lipopolysaccharide (LPS). The DNA content of cell-free supernatants was then assessed by fluorometry. Data are presented as arbitrary fluorescence units (AFU) and represent the mean ± SEM. (B) Representative fluorescence images of LPS- and IL-8-induced NET production by resting and primed neutrophils (*n* = 2). Images were taken at ×20 objective. Arrows point to regions of extracellular DNA. (C) ROS generation by resting (white bars) and TNF-α-primed (black bars) neutrophils in response to 10 ng mL^−1^ IL-8 or 100 ng mL^−1^ LPS stimulation was measured over a 60-min period using luminol-based chemiluminescence. Data are presented as area under the curve (AUC) and represent the mean ± SEM of eight experiments performed on neutrophils obtained from young donors.

Figure 1(A) shows that NET generation, measured as the DNA content of cell-free supernatants, by TNF-α-primed neutrophils was significantly higher than by resting, unprimed neutrophils treated with IL-8 (*P* = 0.001) or LPS (*P* = 0.007), showing that priming enhances NET production. Indeed, when NET formation was studied by fluorescence microscopy, it was evident that in response to both stimuli, primed neutrophils had extruded a greater amount of DNA (Fig. 1B). In addition to enhancing NET production, TNF-α priming significantly increased ROS generation by neutrophils, following IL-8 (*P* < 0.0001) or LPS (*P* < 0.0004) treatment (Fig. 1C).

### Age-associated reduction in IL-8 and LPS-induced NET formation

**Figure 2:**
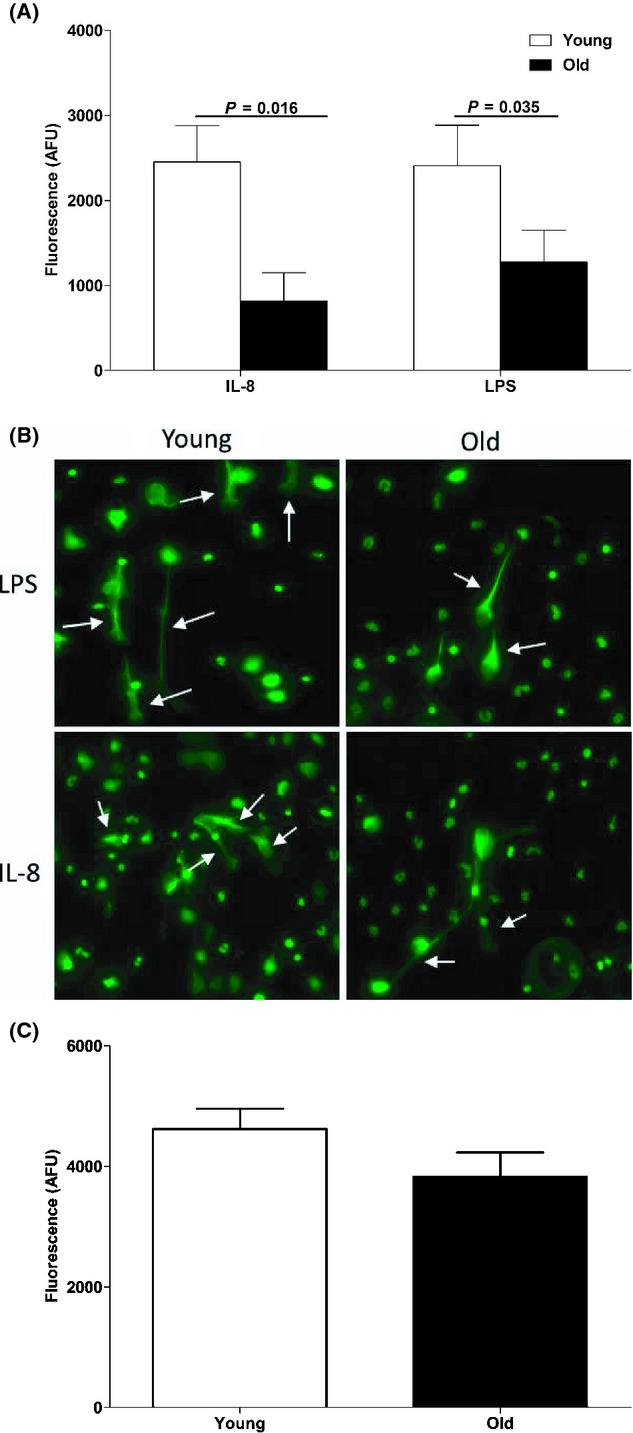
Effect of age on NET production. Neutrophils isolated from young and old donors were primed with 10 ng mL^−1^ TNF-α prior to a 3-h stimulation with 10 ng mL^−1^ IL-8 or 100 ng mL^−1^ lipopolysaccharide (LPS). NET production was assessed either by measuring the DNA content of cell-free supernatants by fluorometry (A) or by immunofluorescence microscopy (B). For microscopy, images were taken at ×20 objective and are representative of two independent experiments. Arrows point to regions of extracellular DNA. For (A), data are presented as arbitrary fluorescence units (AFU) and represent the mean ± SEM. For IL-8 treatment, data are for 9 young and 10 old donors, and for LPS, data are for 8 young and 11 old donors. Results for young adults are depicted in white bars. Results for older adults are presented as black bars. (C) Neutrophils obtained from 10 young and 8 old donors were stimulated for 3 h with 25 nm PMA, after which NET production was assessed by fluorometry. Data are presented as AFU and are mean ± SEM.

To investigate the effect of aging on NET formation, neutrophils isolated from healthy young and healthy older adults were primed with TNF-α and stimulated with either IL-8 or LPS, after which the DNA content of cell-free supernatants was measured. Fluorometric quantification revealed that significantly lower amounts of extracellular DNA were extruded by neutrophils of older adults treated with IL-8 (*P* < 0.02) or LPS (*P* < 0.04) (Fig. 2A), suggesting that aging in healthy adults is associated with reduced NET production. Fluorescence microscopy images confirmed that following IL-8 or LPS stimulation, TNF-α-primed neutrophils from healthy older adults exhibited lower levels of NET formation (Fig. 2B).

Interestingly, no age-associated difference in NET production was observed when neutrophils were treated with PMA (Fig. 2C), a stimulus that bypasses proximal cell membrane receptor signalling events to directly activate protein kinase C, which subsequently phosphorylates and activates NADPH oxidase to generate ROS.

### Effect of age on ROS generation

**Figure 3:**
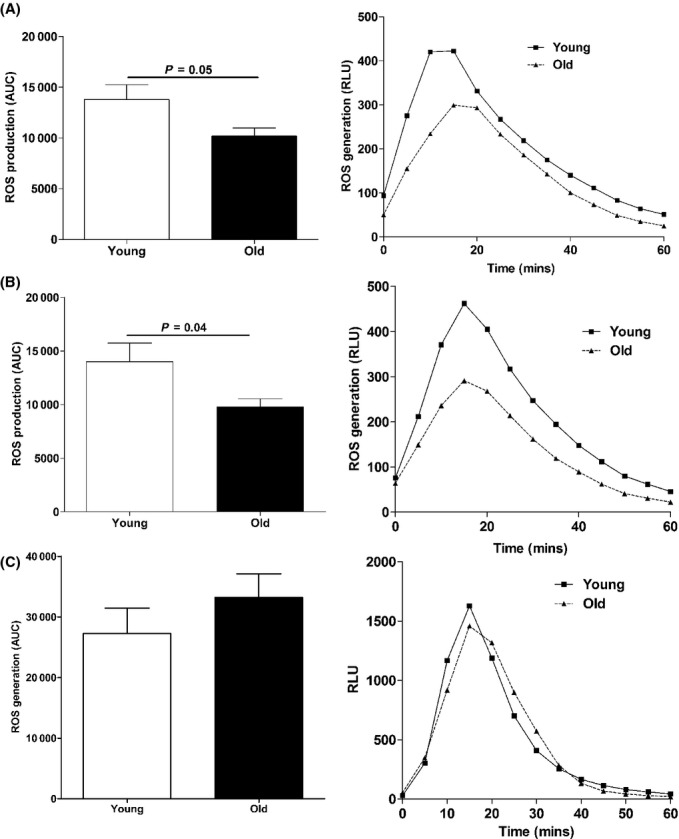
Effect of age on ROS generation. Using luminol-based chemiluminescence, ROS generation by TNF-α-primed neutrophils in response to 10 ng mL^−1^ IL-8 (A) or 100 ng mL^−1^ lipopolysaccharide (LPS) (B) challenge or by resting neutrophils treated with 25 nm PMA (C) was measured over a 60-min period. In panels on the left, ROS generation is presented as area under the curve (AUC) and represents the mean ± SEM for 8 young and 8 old subjects for IL-8 and LPS challenge, and 10 young and 8 old donors for PMA treatment. In panels on the right, representative plots of ROS generation, presented as relative light units (RLU), are shown.

Reactive oxygen species generation is a fundamental event required for NET formation as shown by the inability of neutrophils from patients with chronic granulomatous disease to generate NETs upon stimulation (Fuchs *et al*., 2007; Bianchi *et al*., 2009; Nishinaka *et al*., 2011). Having observed an age-related decline in NET generation, we investigated whether reduced ROS production might underlie this observation. Using a luminol-based chemiluminescence assay that detects the generation of MPO-derived oxygen metabolites, which are responsible for triggering NET formation (Dahlgren & Karlsson, 1999; Nishinaka *et al*., 2011; Palmer *et al*., 2012), we found that when compared to those from younger adults, TNF-α-primed neutrophils from older adults generated significantly less ROS following either IL-8 or LPS challenge (Fig. 3A–B). As observed for NET formation, no age-associated differences were found in ROS production by PMA-treated neutrophils (Fig. 3C).

### Effect of age on IL-8 and LPS receptor expression on the neutrophil surface

The data so far show no age-associated difference in PMA-induced NET or ROS production (Figs 2C and 3C), suggesting that the decline in NET and ROS generation observed following IL-8 and LPS treatment results from an altered proximal signalling response to these stimuli. Neutrophils respond to IL-8 and LPS challenge through their expression of the G protein-coupled receptors CXCR1 and CXCR2, and the pathogen recognition receptor Toll-like receptor 4 (TLR4), respectively. In agreement with a previous study (Fulop *et al*., 2004), we found no age-related differences in the percentage of neutrophils expressing TLR4 or in the surface density of this receptor (Table 1). Analysis of CXCR1 and CXCR2 expression also revealed no difference between neutrophils from young and old subjects in the percentage of receptor-positive cells or median fluorescent intensity values (Table 1).

**Table 1:** Neutrophils were immunostained for expression of TLR4, CXCR1 and CXCR2. Data are shown for the percentage of cells expressing these receptors and their level of expression (MFI) and are mean ± SEM. MFI; median fluorescent intensity

	Percentage positive			Surface density (MFI)		
	Young (*n* = 8)	Old (*n* = 8)	*P* value	Young (*n* = 8)	Old (*n* = 8)	*P* value
TLR4	31.5 ± 6.2	43.3 ± 6.8	0.23	5.3 ± 0.8	7.6 ± 1.2	0.12
CXCR1	99.0 ± 0.37	98.40 ± 0.40	0.30	106.4 ± 9.7	72.2 ± 15.27	0.08
CXCR2	97.7 ± 1.32	97.44 ± 0.67	0.38	72.96 ± 10.89	58.96 ± 9.54	0.23

### Effect of age on p38 MAP kinase activation in TNF-α-primed neutrophils

**Figure 4:**
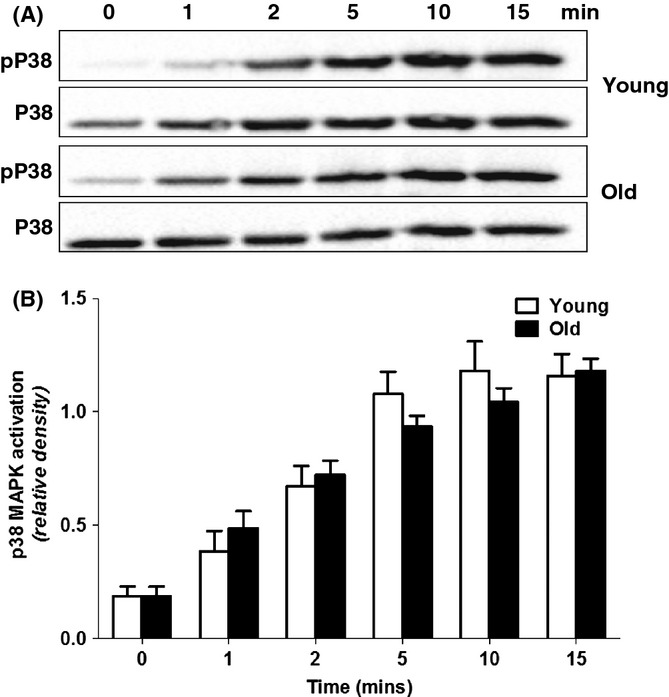
Kinetics of p38 mitogen-activated protein kinase (MAPK) activation in TNF-α-primed neutrophils. Neutrophils isolated from young and old donors were primed over a 15-min time period with 10 ng mL^−1^ TNF-α. At the time points indicated, cell lysates were prepared and p38 activation measured by Western blotting using a phospho-specific anti-p38 antibody. Total p38 expression served as a loading control. (A) Representative Western blot showing p38 activation kinetics in neutrophils isolated from a single young and old donor following TNF-α priming. (B) Densitometric analysis of p38 activation. Data are mean ± SEM for 5 young and 5 old subjects. Results for young adults are depicted in white bars. Results for older adults presented as black bars.

As IL-8 and LPS receptor expression was not affected by aging, we investigated whether an aberrant response to TNF-α priming could have contributed to the age-related decline in LPS- and IL-8 induced NET and ROS generation. Priming neutrophils with TNF-α significantly increases the activity of the mitogen-activated protein kinase (MAPK) p38 (McLeish *et al*., 1998; Dang *et al*., 2006). Once active, this kinase has been shown to augment ROS generation in part by phosphorylating the cytosolic NADPH oxidase subunit p47^PHOX^, triggering its translocation to the plasma membrane (Dang *et al*., 2006). Thus, having observed an age-related decline in ROS production by TNF-α-primed neutrophils and given the integral role of ROS in NET generation (Fuchs *et al*., 2007; Bianchi *et al*., 2009; Palmer *et al*., 2012), we compared by Western blotting the activation (phosphorylation) kinetics of p38 in neutrophils isolated from healthy young and old subjects during our 15-min TNF-α-priming protocol. As shown in Fig. 4, no age-associated differences were found in either baseline or TNF-α-induced p38 phosphorylation (Fig. 4A–B). Using p38 MAPK phosphorylation as a marker of TNF-α-priming efficiency, this result suggests neutrophils from young and old subjects were primed to an equivalent degree prior to secondary stimulation.

### Neutrophil extracellular trap generation in adults with chronic periodontal infection

**Figure 5:**
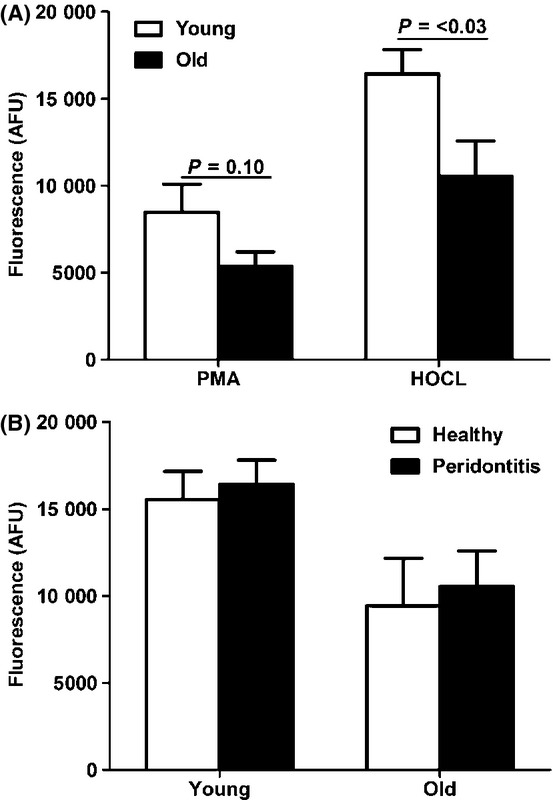
Neutrophil extracellular trap (NET) production by age (< 50 years vs. > 50 years) in periodontitis patients and healthy controls. (A) Peripheral blood neutrophils isolated from young (< 50 years, *n* = 14) and old (> 50 years, *n* = 6) subjects diagnosed with chronic periodontal disease were stimulated with 0.75 mm hypochlorous acid (HOCL) or 50 nm PMA and incubated for 3 h (37°C, 5% CO_2_) prior to quantification of DNA release. (B) Young (*n* = 14) or old (*n* = 6) age-matched healthy controls and periodontitis patient neutrophils (young, *n* = 14; old, *n* = 6) were stimulated with 0.75 mm HOCL and incubated for 3 h (37°C, 5% CO_2_) prior to quantification of DNA release. Data are presented as arbitrary fluorescence units (AFU) and are mean ± SEM.

To ascertain the possible clinical relevance of the age-related reduction in NET generation, we compared NET production induced by PMA or HOCL stimulation between neutrophils obtained from young (mean age 42 ± 4.9 years) and older (mean 57 ± 3.4 years) periodontitis patients, a chronic infectious bacterial disease of humans that increases in prevalence with aging (White *et al*., 2012). Given that periodontitis leads to tooth loss, the ‘older’ dentate patients in this periodontitis substudy had a slightly lower age range when compared to that of the group of healthy older adults already studied. As with the healthy older subjects, in response to PMA treatment, we found no difference in NET production between the two patient groups (Fig. 5A). However, when exposed to the more physiologically relevant stimulus of HOCL, neutrophils derived from older patients released significantly fewer NETs when compared to neutrophils from young periodontitis patients (*P* = < 0.03; Fig. 5A). Interestingly, no differences were observed in absolute NET production between patients and age-matched controls for either stimulus (Fig. 5B).

## Discussion

Here, we demonstrated that priming neutrophils with TNF-α significantly increased NET formation triggered by a secondary IL-8 or LPS stimulus. This observation is in line with a previous *in vitro* study, where priming neutrophils with interferon (IFN)-alpha or IFN-gamma prior to their stimulation with the complement protein C5a significantly enhanced NET production (Martinelli *et al*., 2004), and also concurs with the findings of a recent *in vivo* study, where increased levels of plasma DNA (a marker of NET generation) were recorded in mice that had been treated with granulocyte colony-stimulating factor prior to low-dose LPS challenge (Demers *et al*., 2012). TNF-α priming significantly increased neutrophil ROS production induced by IL-8 and LPS, and given the critical role of ROS in NET formation (Fuchs *et al*., 2007; Bianchi *et al*., 2009; Nishinaka *et al*., 2011), this is one mechanism that could explain how pretreatment with TNF-α augments NET production. However, this may not be the only explanation. In a recent study, Wang and co-workers demonstrated that stimulating human neutrophils for 15 min with TNF-α resulted in the activation of PAD4 (Wang *et al*., 2009). PAD4 catalyses the hypercitrullination of histone H4, a post-translational modification that drives the chromatin decondensation that precedes NET formation (Remijsen *et al*., 2011). Thus, it may be that in TNF-α-primed neutrophils, the process of NET formation is already underway before these cells are subjected to secondary challenge with NET-inducing stimuli.

Physiological aging is associated with an increased incidence of bacterial infections due, at least in part, to the age-related decline in immunity. In mice, aging has recently been shown to result in a significant impairment in NET generation both *in vitro* and *in vivo*, the latter being associated with marked bacteraemia in a model of *Staphylococcus aureus* infection (Tseng *et al*., 2012). Based on this observation, it has been proposed that aberrant NET formation may contribute to the increased incidence of invasive bacterial disease reported by older adults following skin and soft tissue infection (Tseng *et al*., 2012). However, no study had examined the effect of physiological aging in humans on NET generation. Here, we report that when compared to those from younger subjects, TNF-α-primed neutrophils from healthy older adults generated significantly fewer NETs when challenged with IL-8 or LPS, highlighting an additional age-related defect in neutrophil defences.

Reactive oxygen species generation by TNF-α-primed neutrophils following both IL-8 and LPS stimulation also exhibited a significant age-related decline, offering a mechanistic explanation for the reduction in NET formation with age. That aging has a detrimental effect on ROS production by primed neutrophils has previously been reported by Fulop *et al*., who demonstrated neutrophils from older adults were primed less efficiently by the pro-inflammatory cytokine granulocyte macrophage colony-stimulating factor, resulting in significantly lower ROS production upon subsequent stimulation (Fulop *et al*., 2004). Interestingly, we found no age-associated difference in ROS generation or NET formation when neutrophils were challenged with PMA. As a stimulus that bypasses proximal cell membrane receptor signalling events, these results suggest that an aberrant response to TNF-α priming and/or impaired signal transduction following IL-8 and LPS stimulation is responsible for the age-related decline in ROS and NET formation. However, the activation kinetics of p38 MAPK, a signalling element downstream of the TNF-α receptor, were comparable in neutrophils from young and old donors following TNF-α treatment, a finding that is in agreement with the work of Tortorella and colleagues who also reported no age-related difference in p38 MAPK phosphorylation in neutrophils in response to TNF-α priming (Tortorella *et al*., 2004). Our data also show no effect of age on expression of the IL-8 receptors CXCR1 and CXCR2 or the LPS receptor TLR4, confirming a previous observation (Fulop *et al*., 2004). With no difference in receptor expression, our data point to age-related changes to proximal events in receptor signalling as being responsible for the decline in NET and ROS production.

The significance of NETs *in vivo*, with respect to conferring protection in times of pathogenic challenge, has been illustrated in several murine models of infection (Meng *et al*., 2012; Yipp *et al*.,^2012^). Although there are fewer studies in humans, the literature does suggest that reduced NET generation is associated with increased susceptibility to bacterial infections and sepsis (Yost *et al*., 2009). Recently, evidence has emerged challenging the belief that NETs are directly microbicidal, with data suggesting these structures confer protection through pathogen entrapment and neutralization (Menegazzi *et al*., 2012; Parker *et al*., 2012), and via their immunomodulatory properties, which includes a role in T-cell priming (Tillack *et al*., 2012). Thus, an age-related impairment in NET formation may impact negatively upon not only the ability of neutrophils from older adults to confine invading pathogens to the initial site of infection but also their capacity to influence the early phases of an adaptive immune response.

In this study, increased age was associated with reduced NET production in response to HOCL, but not PMA stimulation in periodontally diseased patients. Periodontitis is not a true infection: it has an infectious aetiology, but approximately 80% of the tissue destruction is estimated to arise from a dysregulated inflammatory immune response to plaque biofilm (Grossi *et al*., 1994). Interestingly, no difference was observed in NET production between young or old periodontitis patients and their age-matched healthy controls. Whether reduced NET generation only impacts upon the ability to combat periodontal infections in older adults, in which other aspects of neutrophil function are also compromised (Carneiro *et al*., 2012), remains a possibility. Another plausible explanation for the lack of difference in NET production between periodontitis patients and age-matched controls may relate to the stimulus employed. Hypochlorous acid is the definitive ROS trigger for NET release (Palmer *et al*., 2012), and its addition to blood neutrophils ex vivo may circumvent age-related differences in NET formation arising due to compromised NADPH oxidase activation. Therefore, the potential impact of reduced NET generation upon the course of the disease itself is unclear, as recent evidence from our group has demonstrated an autoimmune signature to periodontitis involving antibodies to citrullinated proteins and their un-citrullinated counterparts (dePablo *et al*., 2013). Citrullinated proteins are a feature of NET production and are associated with autoimmune disease (Kessenbrock *et al*., 2009), and in this respect, it has been proposed that excess NET formation or reduced NET removal from the periodontal tissues may play a role in periodontitis pathogenesis (Cooper *et al*., 2013), similar to other autoimmune diseases (Kessenbrock *et al*., 2009). Thus, the impact of reduced NET production with aging may be disease specific and may differ between diseases of a primarily infectious nature and those where NETs are associated with autoimmune tissue damage.

In summary, we have shown for the first time a propensity for impaired NET release in neutrophils from older adults and an association with reduced ROS production, although reduced NET formation was not further compromised in periodontitis patients.

## Experimental procedures

### Participants

Thirty-nine healthy young (mean age 25.54 ± 4.15 years (mean ± standard deviation), 23 women and 16 men) and 38 healthy old (mean age 69.89 ± 5.34 years, 19 women and 19 men) subjects, who did not report any acute infection and were not taking any medication, were recruited to the study to determine the effect of age on NET generation. In addition, 20 patients (9 women and 11 men) with chronic periodontitis (mean age 42.2 ± 7.1, range 31–61 years), defined as previously reported (Dias *et al*., 2013), and twenty age- and gender-matched controls were enrolled to study NET release by peripheral blood neutrophils in the context of a chronic bacterial infection. Written informed consent was obtained from all participants prior to their enrolment into the study, which was approved by the local research ethics committee (West Midlands Research Ethics Committee number 10/H1208/48).

### Neutrophil isolation and priming

Peripheral blood neutrophils were isolated by Percoll density gradient centrifugation as described previously (Afford *et al*., 1992). Neutrophil purity and viability was determined by Giemsa staining (Diff-Qik; Gentaur Europe, Brussels, Belgium) and trypan blue staining, respectively. Routinely, the purity of neutrophil preparations and cell viability were ≥ 98%. Following isolation, neutrophils were resuspended at a concentration of 1–5 × 10^6^ mL^−1^ in RPMI-1640 media (Sigma-Aldrich, Dorset, UK) supplemented with 2 mm l-glutamine, 100 U mL^−1^ penicillin and 100 μg mL^−1^ streptomycin (hereafter referred to as assay media) or Hank’s balanced salt solution (HBSS) supplemented with calcium and magnesium (hereafter referred to as HBSS^+/+^; Gibco, Invitrogen, Paisley, UK). For priming, neutrophils were treated with 10 ng mL^−1^ TNF-α (Sigma-Aldrich) for 15 min at 37°C in a humidified 5% CO_2_ atmosphere.

### Quantification of extracellular DNA in cell-free supernatants

For the comparison of NET production by neutrophils from young and old volunteers, resting or primed neutrophils (1 × 10^5^ in assay media) were treated with 25 nm PMA (Sigma-Aldrich), 10 ng mL^−1^ IL-8 (R&D Systems, Abingdon, UK), 100 ng mL^−1^ LPS (Sigma-Aldrich) or assay media for 3 h at 37°C in a humidified 5% CO_2_ atmosphere. Postincubation, 1 U mL^−1^ of micrococcal nuclease (MNase; Sigma-Aldrich) and 1 μm of the cell-impermeable DNA binding dye SYTOX Green (Life Technologies, Paisley, UK) were added to cells in order to digest and stain extracellular DNA, respectively. After a 10-min incubation in the dark at room temperature (RT), cells were pelleted and extracellular DNA content was determined in the cell-free supernatant by measuring fluorescence in a Fluoroskan Ascent fluorometric plate reader (Labsystems Affinity Sensors, Cambridge, UK) or a BioTek® Synergy 2 fluorometric plate reader (NorthStar Scientific Ltd, Leeds, UK), at an excitation wavelength of 485 nm and emission at 530 nm. Experiments were performed in quadruplicate, and background fluorescence values of neutrophils treated with assay media alone were subtracted from test samples.

In the periodontitis study, 1 × 10^5^ neutrophils in RPMI 1640 media supplemented with 1% glutamine were added to a preblocked (syringe-filtered 1% BSA) 96-well plate and stimulated with either 50 nm PMA or 0.75 mm HOCL and incubated for 3 h (37°C, 5% CO_2_). Post-treatment, DNA release was determined as above. No TNF-α priming was employed in this study, as peripheral blood neutrophils from periodontitis patients are already primed within the circulation by plasma cytokines (Wright *et al*., 2008; Dias *et al*., 2010).

### Assessment of NET formation by immunofluorescence microscopy

Visualization of NETs by immunofluorescence was performed using an adapted version of the protocol described by Brinkmann *et al*. (Brinkmann *et al*., 2010). Briefly, resting or primed neutrophils (2 × 10^5^ in assay media) were seeded onto 13-mm circular glass coverslips (VWR International, Leicestershire, UK) and incubated for 30 min at 37°C in a 5% CO_2_ atmosphere to allow for cell adherence. Postincubation, neutrophils were stimulated with PMA (25 nm), IL-8 (10 ng mL^−1^), LPS (100 ng mL^−1^) or assay media for 3 h (37°C, 5% CO_2_), after which cells were fixed in 2% paraformaldehyde for 30 min at 37°C. Once fixed, coverslips were subjected to three 5-min washes in PBS at RT, after which cells were permeabilized in 0.1% Triton x-100 (Sigma-Aldrich) for 1 min at RT. After a single 5-min wash in PBS, samples were stained with 1 μm SYTOX Green for 5 min, followed by a 5-min wash in PBS. Once stained, specimens were mounted in fluoromount medium onto glass microscope slides (VWR International) and visualized using a LEICA DMI 6000 B microscope (Leica Microsystems, Milton Keynes, UK) at ×20 objective. The criterion used to define a NET was extracellular DNA in the form of a ‘line’ or ‘cloud’.

### Measurement of ROS generation

Reactive oxygen species generation was assessed by luminol-amplified chemiluminescence. Resting or primed neutrophils (1 × 10^5^ in HBSS^+/+^) were dispensed into wells of a 96-well white-bottomed flat plate (Corning) that contained 1 μm luminol (pH 7.3; Sigma-Aldrich). Cells were then stimulated with LPS (100 ng mL^−1^), IL-8 (10 ng mL^−1^), PMA (25 nm) or vehicle control, after which ROS generation was assessed at 1-min intervals for 60 min using a Berthold Centro LB 960 luminometer (Berthold Technologies, Hertfordshire, UK). Experiments were performed in quadruplicate, with ROS production measured as relative light units (RLU) and calculated as area under the curve (AUC).

### Measurement of p38 MAP kinase activation by Western blotting

Freshly isolated resting neutrophils (5 × 10^6^ mL^−1^ in assay media) were treated with10 ng mL^−1^ TNF-α for 0–15 min at 37°C in a humidified 5% CO_2_ atmosphere. Poststimulation, neutrophils were pelleted by centrifugation (1500 *g*, 2 min, 4°C), supernatants discarded and cell lysates prepared by the addition of hot sodium dodecyl sulphate (SDS) sample buffer [4% SDS (v/v), 0.1 m dithiothreitol, 20% glycerol (v/v), 0.0625 m Tris–HCl and 0.004% bromophenol blue (w/v)]. Lysates were separated on 10% SDS polyacrylamide gels and proteins transferred to polyvinylidene difluoride membranes (Scientific Laboratory Supplies, Yorkshire, UK). To prevent nonspecific binding, membranes were incubated with 5% bovine serum albumin (BSA) in Tris-buffered saline (TBS; 200 mm Tris (pH 7.5), 1.5 m NaCl) containing 0.1% Tween-20 (TBST) for 1 h at RT, before being probed overnight at 4°C with a rabbit anti-human phospho-p38 antibody (diluted 1:1000 in TBST containing 2.5% BSA; Cell Signalling Technology, Hertfordshire, UK). Postincubation, blots were washed in TBST and incubated for 1 h at RT with a goat anti-rabbit secondary antibody conjugated to horseradish peroxidase (HRP; diluted 1:4000 in TBST; GE Healthcare, Buckinghamshire, UK). Horseradish peroxidase activity was detected using enhanced chemiluminescence (Geneflow, Staffordshire, UK). To confirm equal loading of proteins, blots were stripped with stripping buffer (10% (w/v) SDS, 62.5 mm Tris–HCl (pH 6.8), 0.08% (v/v) ß-mercaptoethanol) for 45 min at 50°C, washed for 2 h in distilled H_2_O and blocked for 1 h at RT in 5% BSA/TBST, before being probed overnight at 4°C with a p38 antibody (diluted 1:2000). Densitometry was performed using the National Institute of Health ImageJ software package.

### Measurement of surface receptor expression

Freshly isolated neutrophils (1 × 10^5^) were stained with 2 μg mL^−1^ anti-CXCR1-FITC (R&D Systems; clone 42075), 3 μg mL^−1^ anti-CXCR2-FITC (R&D Systems; clone 48311) or 5 μg mL^−1^ anti-TLR4-APC (clone HTA125; eBioscience, Hatfield, UK) antibodies or their relevant concentration-matched isotype controls for 20 min on ice. Postincubation, cells were washed once in PBS/1% BSA, resuspended in 100 μL PBS and transferred to polypropylene FACS tubes. Flow cytometry was conducted using a CyAn_ADP_™ bench-top cytometer (Dako, Cambridgeshire, UK) with 5000–10 000 neutrophils acquired for analysis, which was performed using Summit v4.3 software (Dako Colorado Incorporation, Fort Collins, CO, USA). The percentage of antigen-positive neutrophils was recorded along with the corresponding median fluorescence intensity (MFI) values.

### Statistical analysis

Statistical analyses were performed using GraphPad Prism® software (GraphPad Software Ltd, San Diego, CA, USA). Data distribution was examined using the Kolmogorov–Smirnov test. For data that followed a normal distribution, unpaired and paired student *T*-tests were performed to assess differences between two independent groups or matched paired samples, respectively. A repeated measures anova was used to compare more than two matched observations. For non-normally distributed data, a Mann–Whitney *U* test was used. Data are presented as mean ± standard error of the mean (SEM). The minimum level of confidence at which results were considered statistically significant was *P* ≤ 0.05.
